# Melanoma microsatellites exhibit a metastatic signature by spatial transcriptomics and overexpress mediators of immune evasion

**DOI:** 10.1007/s00428-025-04182-7

**Published:** 2025-07-28

**Authors:** Simon J. Warren, Gloria R. Xue, Hong-Ming Zhou, Ahmed K. Alomari, Matthew J. Turner

**Affiliations:** 1 Departments of Dermatology and Pathology, University of Michigan, 36-1221-15, 2800 Plymouth Rd, Building 35, Ann Arbor, MI 48109-2800 USA; 2 Department of Dermatology, Indiana University, Indianapolis, IN USA; 3 Department of Pathology, Indiana University, Indianapolis, IN USA

**Keywords:** Spatial transcriptomics, Melanoma microsatellites, Metastasis, Immune evasion

## Abstract

Microsatellites in melanoma are physically separate from the primary tumor, are hypothesized to arise by lymphovascular invasion similar to other metastases, and *have not been previously studied at the molecular level*. We compared gene expression in microsatellites with the matched primary melanoma in two patients using spatial transcriptomics. We identified recurrently altered genes in microsatellites contributing to vascular invasion (ITGA4, MCAM), survival in the circulation (CXCL8, PDGFRB, CDH1), vascular exit (ITGB2), survival at metastatic sites (C3, TGBFI), matrix remodeling (MMP9, VCAN, FN1, BGN), and angiogenesis (EMILIN2). Gene set enrichment analysis confirmed a metastatic signature in comparison to genes upregulated and downregulated in distant melanoma metastases, and identified pathway enrichment for NFKB, CDH1, and ZEB1 signaling. Microsatellites exhibited increased host immune responses and four recurrently overexpressed mediators of immune evasion were present (PAEP, GDF15, CD74, HLA-DRA) implying selection pressure from the increased immune response. Seven additional non-recurrent mediators of immune evasion were identified in microsatellites, overexpressed up to 58-fold in microsatellites. PAEP was 275-fold and 25-fold overexpressed, respectively, in microsatellites in two cases. We studied PAEP protein expression by immunohistochemistry in a larger group of 12 and found overexpression in microsatellites in five of 12 patients. PAEP was detected by immunohistochemistry in a microsatellite and also in a subclone within its primary, raising the possibility that the immunostain identifies the subclone in the primary responsible for the microsatellite. These preliminary findings suggest that melanoma microsatellites are true metastases at the level of gene expression, accounting for the sharply increased risk of disease progression in patients with microsatellites.

**Supplementary Information:**

The online version contains supplementary material available at 10.1007/s00428-025-04182-7.

## Introduction

Melanoma is now the third most common cancer diagnosed, with 192,000 new cases in the USA in 2019, and metastatic melanoma accounts for nearly all deaths. However, a major gap in our understanding of melanoma is the specific molecular changes that underpin the evolution of metastatic melanoma from primary melanoma. Previous studies have found recurrent *somatic* mutations in DNA in the early evolution of primary melanoma from precursor lesions such as nevi, but no recurrent somatic mutations in DNA have been identified in the evolution of metastatic melanoma from primary melanoma [1, 2]. By contrast, studies at the level of RNA comparing primary and metastatic melanoma have identified many differentially expressed genes (DEGs), ranging from 308 to ~ 1300 genes [3–5]. A single case studied where the primary melanoma and metastasis were derived from the same patient identified ~ 200 differentially expressed genes [6]. These findings suggest that changes in RNA expression rather than somatic mutations in DNA are important for this stage in melanoma evolution. Given the large number of DEGs identified in these studies, it has been hard to dissect out which genes are functionally relevant. Further, the large numbers of DEGs identified in these studies likely reflect the complex evolution of metastatic melanoma from primary melanoma, which requires the melanoma to adapt to multiple sequential selection pressures. These include overriding cell cycle control and senescence mechanisms, avoiding the attention of the immune system, invasion of the dermis, vascular invasion, anoikis resistance (survival in the circulation), exiting the circulation (diapedesis), and colonization at varied secondary tumor sites where melanocytes would not normally be present [7, 8].

Further, the use of unmatched primary melanomas and metastatic melanoma limits the ability to gain useful information from these studies, since each individual melanoma likely is on a different evolutionary trajectory and the changes in RNA expression that distinguish an individual melanoma from its own metastasis cannot be discerned. The bulk sequencing methods used in these studies also make it hard to distinguish genes that are differentially expressed by the tumor primary vs. metastasis from those that are differentially expressed by skin stromal cells vs. stromal cells at the site of distant metastasis.

We therefore studied the evolution of microsatellite disease by comparing RNA expression in microsatellites to their adjacent matched primary melanoma using spatial transcriptomics. Since the primary and microsatellite are present in the same tissue stroma, the issue of genes that are differentially expressed due to different tissue stroma does not apply.

A microsatellite is defined as a stage in melanoma progression that is physically separated from the primary melanoma, but in the same tissue. Microsatellites are hypothesized to arise by a process that includes lymphovascular invasion, survival for a short time in the circulation, exit from the lymphovascular space, and then proliferation at a nearby site in the same tissue [9]. Microsatellites are classified with a higher pathologic stage of N1c or N2c in the AJCC pathologic staging of melanoma [9], and that correlates with a worse clinical outcome [10–14]. In a recent study, 69 cases of melanoma with microsatellites had a 5-year disease-free survival of 21% compared to 73% in the group of *matched* melanoma controls lacking microsatellites [14].

Spatial transcriptomics allows the study of small increments of tumor evolution that are present in a single tissue section. In this study, we focused on melanoma microsatellites, which have not previously been studied at the molecular level. We identified a number of genes that are recurrently overexpressed and recurrently lost that facilitate immune evasion, matrix remodeling, angiogenesis, vascular invasion, anoikis resistance/survival in the circulation, diapedesis/exit from the circulation, and survival at metastatic sites. We validated our findings in the most highly differentially expressed gene, PAEP (progestogen-associated endometrial protein) using immunohistochemistry in a larger group of 12.

## Results

**Fig. 1:**
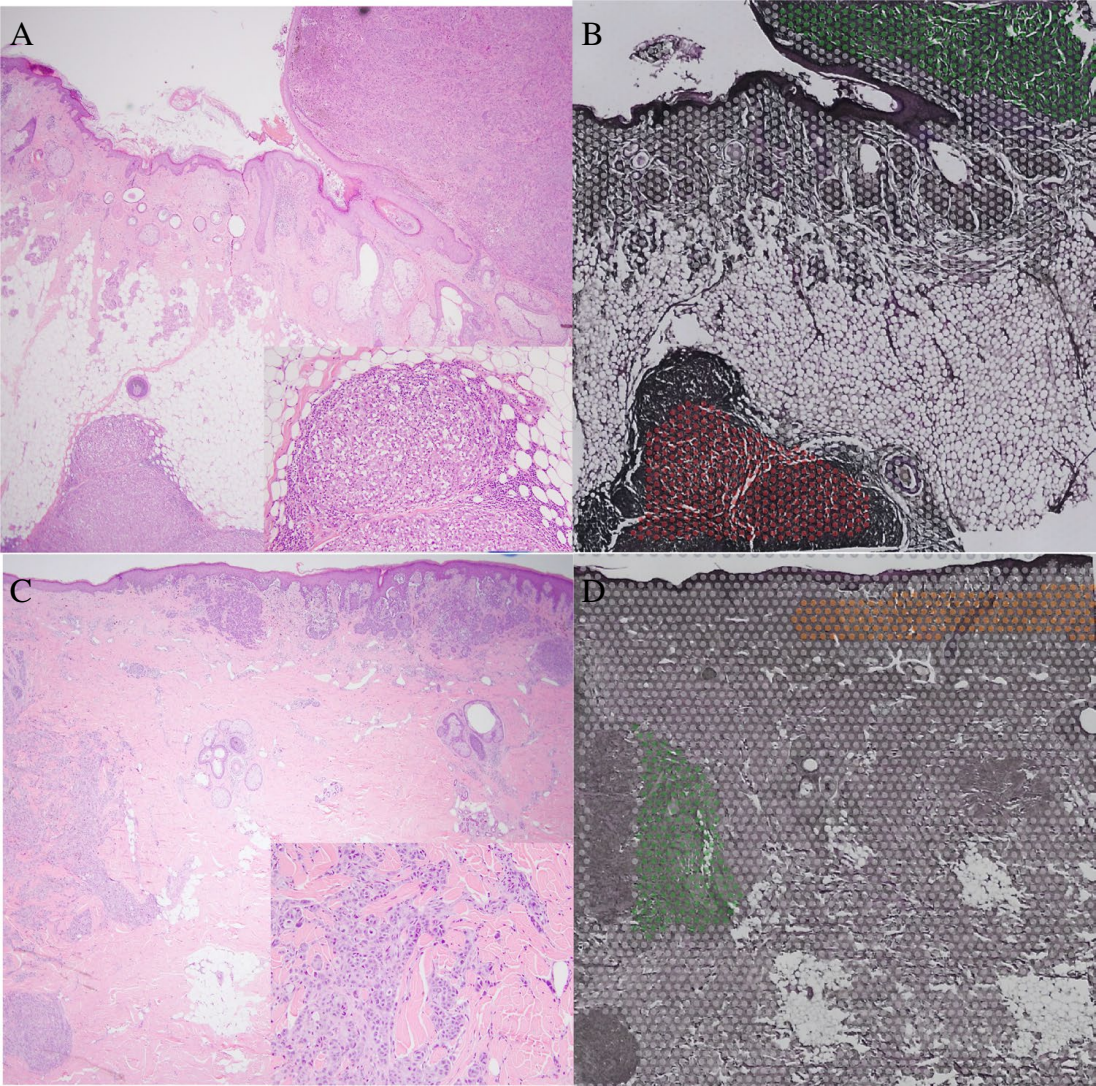
Experimental approach and selection of areas of primary melanomas and microsatellites for analysis. **A** Case 1, original H&E showing primary melanoma (upper right) and microsatellite (lower left), 40 ×. Inset: microsatellite with dense lymphocytic host response, 200 ×. **B** Case 1 showing areas of tumor selected for analysis overlaid on H&E image of section analyzed. Primary melanoma: green. Microsatellite: red. **C** Case 2, H&E showing primary melanoma (upper right) and microsatellite (lower left), 40 ×. Inset: microsatellite lacking dense lymphocytic host response, 200 ×. **D** Case 2, areas of tumor selected for analysis overlaid on H&E image of section analyzed. Primary melanoma: orange. Microsatellite: green. White dots in **B**, **D** are individual 55 µm RNA probe capture areas

**Fig. 2:**
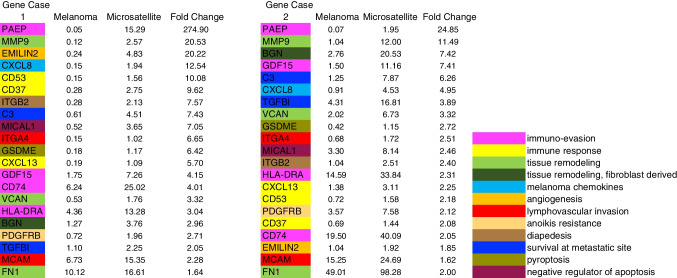
Normalized expression for selected *recurrent* overexpressed genes, ranked by fold increase in microsatellite over primary melanoma. Left panel: case 1. Right panel: case 2. Genes are annotated by function (bottom right)

Areas of the primary melanoma and microsatellite were selected for comparison in Loupe Browser software (10 × Genomics) using histologic criteria from the superimposed H&E image (Fig. 1). There were 420 genes that more than doubled their level of expression in the microsatellite in case 1 and 345 genes in case 2. There were 66 recurrent genes shared by the two cases that more than doubled expression in the microsatellite. Figure 2 shows selected *recurrent* overexpressed genes for cases 1 and 2, ranked by fold increase in the microsatellite over the primary melanoma, and annotated according to function.

Recurrent overexpressed genes in microsatellites that mediate *matrix invasion and tissue remodeling* include MMP9, VCAN, and FN1. All three have reported roles in melanoma [15, 16]. MMP9 is 20.5-fold and 11.5-fold overexpressed in the microsatellites from cases 1 and 2, respectively. MMP9 is involved in the degradation of the ECM, but is also involved in neo-angiogenesis, cell migration, and formation of melanoma metastases [15].

Recurrent genes identified that produce *melanoma associated chemokines* include CXCL8, which is 12.5-fold and fivefold overexpressed in the microsatellites from cases 1 and 2, respectively. In melanoma, CXCL8 is a multifunctional cytokine that promotes neo-vascularization, activates MMP-2, and enhances anoikis resistance [17].

Recurrent genes identified that mediate *lymphovascular invasion* include ITGA4 (α4 integrin) and MCAM. ITGA4 was 6.65-fold overexpressed in the microsatellite in case 1 and 2.5-fold overexpressed in case 2. ITGA4 expression on melanocytes allows adhesion to vascular cell adhesion molecule 1 (VCAM-1) on lymphatic endothelial cells and has been shown to facilitate melanoma lymph node metastases in a mouse model [18]. MCAM was recurrently overexpressed ~ twofold and mediates adhesion to endothelium [19]. EMILIN2 was recurrently overexpressed and stimulates angiogenesis [20].

Recurrent genes that mediate anoikis resistance/*survival in the circulation* include CXCL8 [21, 22] and PDGFRB [23]. CDH1 (E-cadherin) was 30-fold and 14-fold reduced in the microsatellites from cases 1 and 2, respectively. CDH1 loss also enables anoikis resistance [23, 24].

Recurrent overexpressed genes that mediate diapedesis (*exit from the circulation*) include ITGB2 (IntegrinB2). This dimerizes with integrin αL to form leukocyte-function-associated antigen 1 (LFA-1), which binds intercellular adhesion molecules (ICAMs) on endothelial cells as part of the process of diapedesis [25]. High differential expression of IntegrinB2 relative to the initiating primary melanoma has been reported in melanoma brain metastases in a mouse model [26].

Recurrent genes that mediate *survival at metastatic sites* include C3 and TGFBI. C3 was recurrently overexpressed 7.4-fold and 6.25-fold in the microsatellites. Upregulation of C3 produced by cancer cells themselves has been reported to promote leptomeningeal metastasis by disrupting the integrity of the blood-cerebrospinal fluid barrier, allowing cancer cells to access mitogens and nutrients at the metastatic site [27]. Blockade of C3 signaling via the C3a receptor also blocks leptomeningeal metastases in a mouse model [27]. TGFBI is a secreted extracellular matrix protein that has been shown to be important for the growth of melanoma distal metastases after intravenous injection in a mouse model but not for initial dissemination or extravasation to the metastatic site [28].

Recurrent overexpressed genes identified that have been reported to mediate *immune evasion* included PAEP [29, 30], GDF15 [31], CD74 [32, 33], and HLA-DRA [33]. PAEP is 275-fold and 25-fold overexpressed in the microsatellites from cases 1 and case 2, respectively. PAEP (progestagen-associated endometrial protein) is normally produced by the placenta and endometrium and has been reported to suppress T-cell function in melanoma [29, 30]. Recurrent host immune response genes detected in microsatellites include CXCL13, CD53, and CD37 [34, 35].

**Fig. 3:**
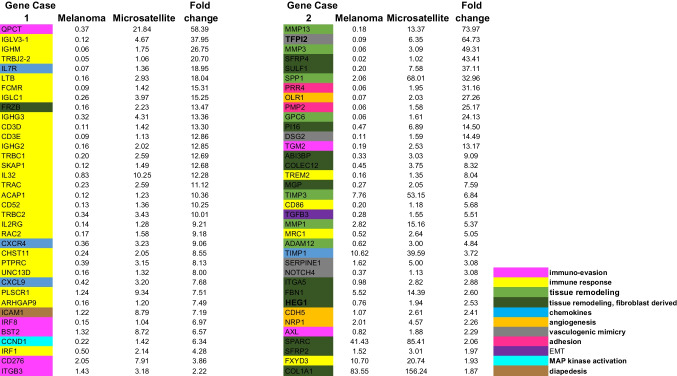
Normalized expression for selected *non-recurrent* overexpressed genes, ranked by fold increase in microsatellite over primary melanoma. Left panel: case 1. Right panel: case 2. Genes are annotated by function (bottom right)

**Fig. 4:**
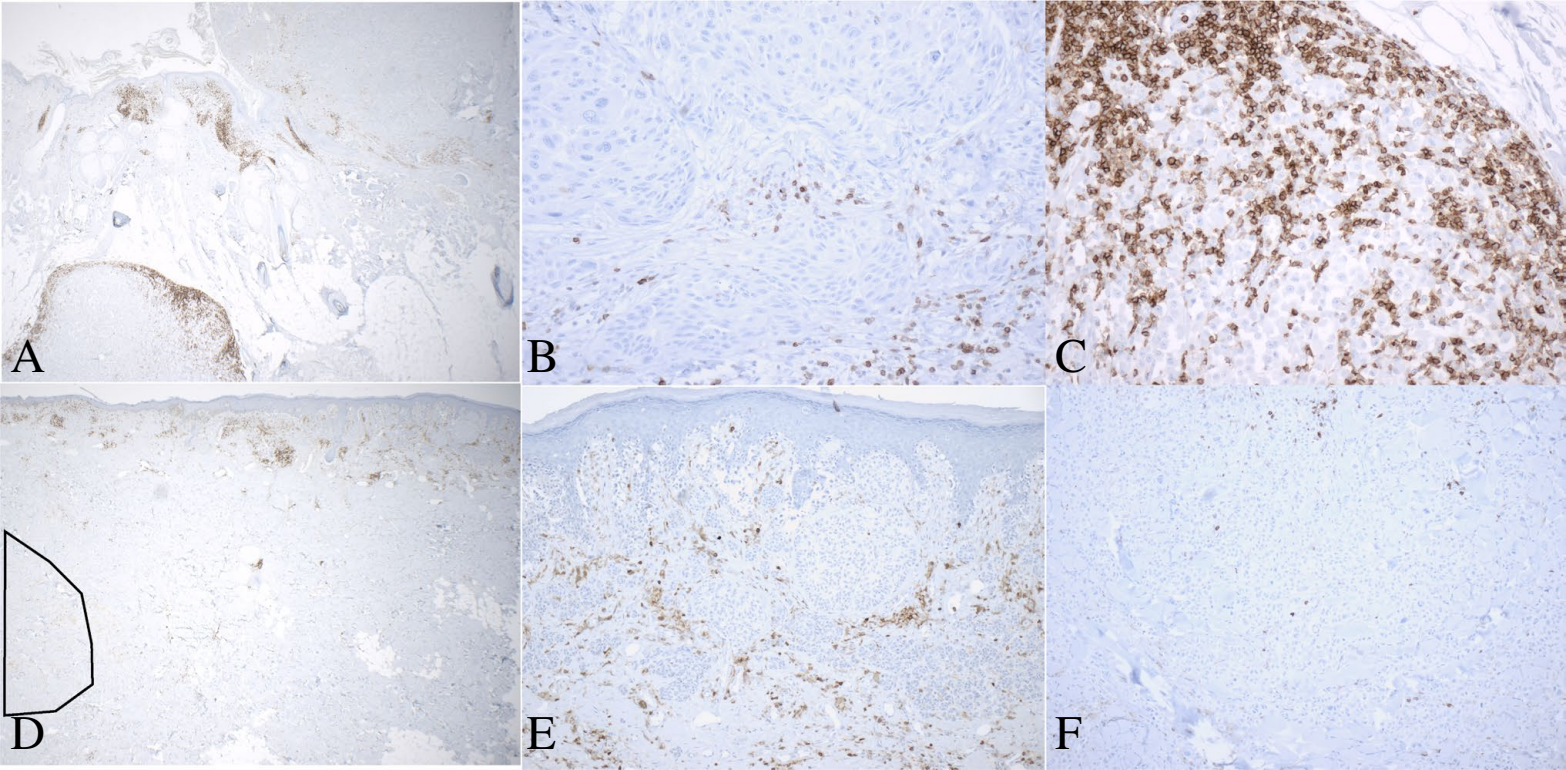
Leukocyte common antigen immunostain in primary melanomas and their microsatellites. **A**, **B**, **C** Case 1, microsatellite associated with high immune response. **A** Primary melanoma (top right) and microsatellite, bottom left, 20 ×. **B** Primary melanoma, 200 ×. **C** Microsatellite 200 ×. **D**, **E**, **F** Case 2, microsatellite associated with low immune response. **D** Primary melanoma (top right) and microsatellite, bottom left, outlined in black, 20 ×. **E** Primary melanoma, 200 ×. **F** Microsatellite 200 ×

We also studied the most overexpressed genes present in one of our cases but not the other (Fig. 3). In case 1, there were five additional mediators of *immune evasion* (QPCT, IRF8, BST2, CD276, and ITGB3). QPCT was 58-fold overexpressed; its isoform QPCTL has been reported to activate CD47 on tumor cells, leading to inhibition of phagocytosis by immune cells [36]. QPCT has been reported to be overexpressed in melanoma and to complement the activity of QPCTL [37]. In case 2, only two additional mediators of immune evasion were identified in the microsatellite, TGM2 and AXL. This corresponded to the presence of many more markers of host immune response in case 1 relative to case 2 (Fig. 3, annotated in yellow). The more pronounced host-immune response in the microsatellite from case 1 was also present on H&E images (Fig. 1A inset and C inset) and on leukocyte common antigen (LCA) immunostain (Fig. 4). Taken together, these findings suggest that in the microsatellite from case 1, the greater host immune response provides more selection pressure on gene expression, resulting in overexpression of mediators of immune evasion relative to case 2.

By contrast in case 2, there were seven additional *matrix remodeling* genes overexpressed (MMP13, MMP3, SPP1, GPC6, TIMP3, MMP1, ADAM12), with MMP13 74-fold overexpressed in the microsatellite. There were also 12 markers of tissue remodeling derived from fibroblasts overexpressed (Fig. 3). Four markers of *vasculogenic mimicry* were overexpressed in the microsatellite in case 2: TFPI2, DSG2, SERPINE1, and NOTCH4. TFPI2 was overexpressed 65-fold in case 2 and has been reported to be essential for vasculogenic mimicry in melanoma [38]. DSG2 was overexpressed 14.5-fold in case 2 and also enables vasculogenic mimicry [39] a mechanism by which melanoma cells increase their blood supply and are directly connected to the circulation, permitting vascular invasion [39, 40]. SERPINE1 encodes a protease which provides anticoagulation, which is critical for vasculogenic mimicry in breast cancer [39]. NOTCH4 activity is required for vasculogenic mimicry via the NODAL pathway [41].

PRR4 (Nectin4) is overexpressed 31-fold in the microsatellite from case 2. It mediates *homotypic cell adhesion* [42] and takes part in adherens junctions with N-cadherin. Overexpression has been reported in cancer progression in a range of cancers [43]. There is also an FDA-approved Nectin4 antibody–drug conjugate that is currently being used in bladder and breast cancer [43].

**Fig. 5:**
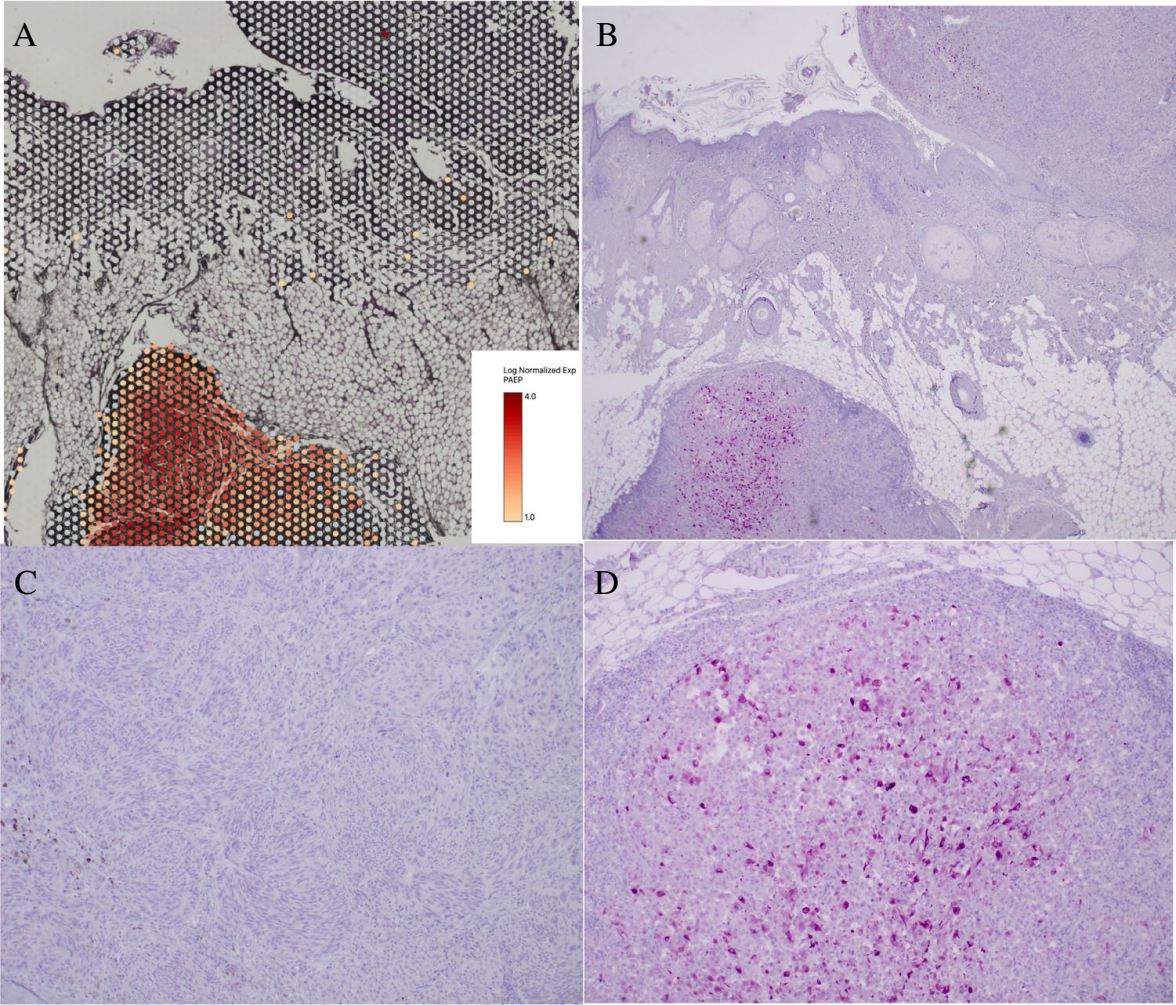
PAEP RNA and protein expression in primary melanoma and microsatellite. **A** Case 1, PAEP RNA expression in microsatellite (bottom left) relative to its primary melanoma (top right), superimposed on H&E image of section analyzed. Inset: PAEP expression, log2 scale. Dots represent individual 55-µm RNA probe capture areas. **B** Case 1, adjacent tissue section, PAEP immunostain in microsatellite (bottom left) relative to its primary melanoma (top right), magenta chromogen, 40 ×. **C** PAEP immunostain, primary melanoma, 200 ×. **D** PAEP immunostain, microsatellite, 200 ×. Note dense immune response, visible at periphery of microsatellite

**Fig. 6:**
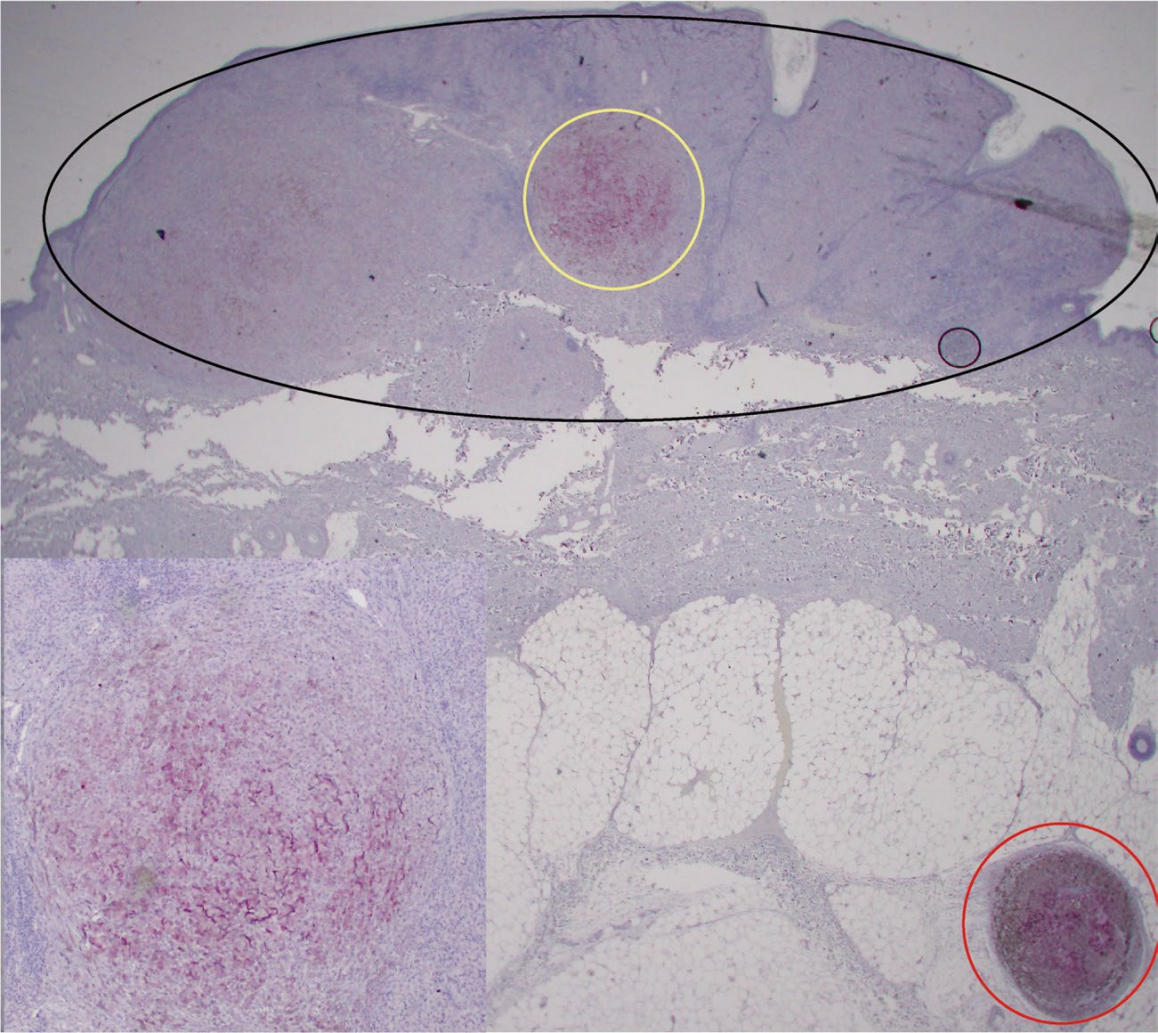
PAEP immunostain is positive in the microsatellite and a subclone in the primary melanoma. Primary melanoma (black oval) with microsatellite (red circle). Microsatellite expresses PAEP as does a small subclone (yellow circle) within the primary melanoma. Magenta chromogen, 20 ×. Inset: subclone in primary melanoma, 100 ×

We confirmed *protein expression by means of immunohistochemistry* for the most overexpressed gene, PAEP. Figure 5 shows PAEP RNA expression in the microsatellite from case 1 relative to its primary melanoma visualized in Loupe Browser (panel A). Panels B, C, and D show PAEP protein overexpression in the microsatellite relative to its originating melanoma. It is noted that dense immune response is visible at the periphery of the microsatellite in panel D. *We studied a larger group of 12 cases by immunohistochemistry and showed PAEP protein overexpression in microsatellites in five of 12 cases.* Intriguingly, screening of additional cases with the PAEP immunostain also identified a histologically inapparent sub-clone within a primary melanoma that shares PAEP expression with the microsatellite and could therefore represent the sub-clone of origin for the microsatellite (Fig. 6). We also studied five sentinel nodes from a single patient using the PAEP immunostain and identified reactivity for PAEP in two of five.

**Fig. 7:**
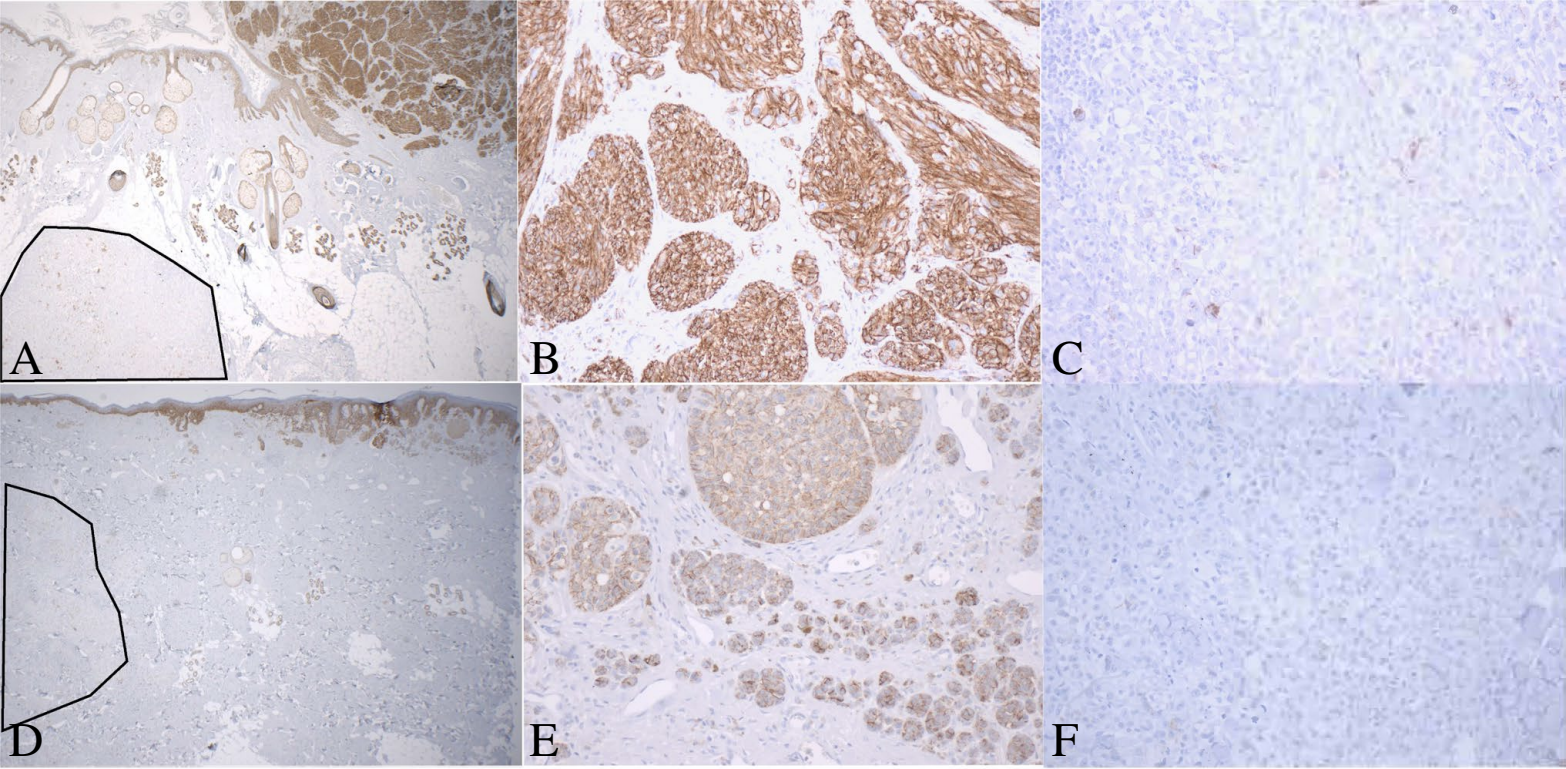
E-cadherin immunostain is positive in the primary melanomas and lost in the microsatellites. **A**, **B**, **C** Case 1: **A** primary melanoma (top right) and microsatellite, bottom left (black outline), 20 ×. **B** Primary melanoma, 200 ×. **C** Microsatellite, 200 ×. **D**, **E**, **F** E-cadherin immunostain, case 2. **D** Primary melanoma (top right) and microsatellite, bottom left (black outline), 20 ×. **E** Primary melanoma, 200 ×. **F** Microsatellite, 200 ×. DAB chromogen

We evaluated protein expression for CDH1 (E-cadherin), which is 30-fold and 14-fold reduced in the microsatellites from cases 1 and 2, respectively. Loss of E-cadherin mediates anoikis resistance. We confirmed loss of protein expression in both microsatellites relative to their primary melanoma (Fig. 7).

Since four RNA markers of vasculogenic mimicry were overexpressed in the microsatellite from case 2 relative to its primary melanoma, we evaluated case 2 for histologic evidence of vasculogenic mimicry using combined CD31/PAS stain. Using this combination stain, normal capillaries stain with CD31 while vasculogenic mimicry is negative for CD31 and positive using PAS histochemical stain [44]. Vasculogenic mimicry was confirmed in the microsatellite from case 2 but not in its primary melanoma, as predicted by the RNA expression data (see Supplementary Figure S1).

We performed *gene set enrichment analysis* with GSEA 4.4.0 [45, 46] using all upregulated and downregulated genes in microsatellites in cases 1 and 2. Compared to a set of genes differentially expressed in 22 melanoma distant metastases relative to 19 primary melanomas [3], we identified significant enrichment for both upregulated and downregulated genes: case 1, upregulated *p* = 0.003, downregulated *p* = 0.0; case 2, upregulated *p* = 0.003, downregulated *p* = 0.0. Of 46 upregulated genes in the gene set from Jaeger et al. [3], 31 were upregulated in case 1 and 32 in case 2. Of 260 downregulated genes in the gene set from Jaeger et al. [3], 146 were downregulated in case 1 and 209 in case 2 (see Supplementary Figure S2). Recurrent genes of interest in this gene set included SPP1, FN1, CDH1, and PTPRZ1.

Using gene set enrichment analysis, we also identified recurrent *pathway enrichment* for NFKB signaling in the genes overexpressed in our microsatellites, in comparison to NFKB pathway genes identified in cultured melanoma cells by small molecule inhibition of NFKB [47]. Case 2 was significantly enriched (*p* = 0.011) and case 1 did not reach significance (*p* = 0.11); however, recurrent overexpressed genes present in this pathway in both cases included MMP9, CXCL8, C3, and FN1 (see Supplementary Figure S3). We also uncovered significant downregulation of CDH1 targets in our microsatellites in comparison to CDH1 targets identified by RNAi knockdown (see Figure S3, panels C, D) [48]. Both cases were significantly enriched, case 1: *p* = 0.0 and case 2: *p* = 0.0. There were 195 CDH1 targets present in case 1 and 296 in case 2. ZEB1 is a key inhibitor of transcription upstream of CDH1, and we identified significant downregulation of ZEB1 targets compared to ZEB1 targets identified by knockdown of ZEB1 in breast cancer cells [48]. Case 1: *p* = 0.03, case 2: *p* = 0.0 (see figure S3, panels E, F). There were 22 downregulated Zeb1 targets present in case 1 and 25 in case 2. Recurrent genes under-expressed in microsatellites in this pathway included CDH1, CDH3, GJB3, DSP, and PCDH7, which all have roles in cell–cell adhesion [48]. NFKB, CDH1, and ZEB1 are therefore potential master regulators for genes differentially expressed in melanoma microsatellites.

## Discussion

Melanoma microsatellites confer a sharply worse prognosis relative to matched melanomas without microsatellite disease [14] and have not been studied at the molecular level prior to this study. We have studied RNA expression changes that distinguish primary melanomas from their adjacent microsatellites across the whole transcriptome. We have identified differentially expressed genes that are recurrent, biologically relevant, and surprisingly highly overexpressed up to 275-fold in the microsatellites. The changes we have identified parallel metastatic melanoma evolution in general, and support the hypothesis that microsatellites, like other melanoma metastases, arise from a process that involves tissue remodeling, lymphovascular invasion, survival in the circulation, exit from the circulation, proliferation at a distant site, and immune evasion. Our findings by gene set enrichment analysis of significant enrichment for genes differentially expressed in *distant* melanoma metastases support this hypothesis.

Each melanoma and microsatellite are matched because they come from the same patient and same tissue. This allows the primary melanoma to act as a close control for its own microsatellite. Because the melanoma and microsatellite are in the same tissue, differentially expressed genes resulting from stromal cell contamination in a different tissue environment are excluded from evaluation. We have validated our findings for CDH1 and PAEP by immunohistochemistry and demonstrated PAEP overexpression in microsatellites in five of 12 patients.

Our approach has identified 420 genes where expression more than doubles in the microsatellite in case 1 and 345 in case 2. Expression more than halved in 524 genes in case 1 and 421 genes in case 2. There were 66 recurrent overexpressed genes present across both data sets. Lesser numbers of recurrent genes where expression more than halved were identified. These genes have been screened for biologic plausibility as “metastasis genes” based on previously reported gene expression and experimental data. These findings demonstrate the high degree of plasticity possible and required in melanoma gene expression to adapt to a different host environment, even across a sub-millimeter space from primary melanoma to microsatellite in the same tissue.

There are several additional novel findings. We identified recurrent overexpression of four mediators of immune evasion in microsatellites, and an additional seven non-recurrent but overexpressed up to 58-fold in microsatellite disease. *Notably*, *this list does not include the PDL1-PD1 pathway in the microsatellites we studied*. We found increased markers of host response in microsatellites, which presumably act as selection pressure on melanoma cells to express mediators of immune evasion. Comparison of case 1 with case 2 suggests that a more intense host immune response in case 1 may provide selection pressure for greater expression of mediators of immune evasion. Screening of additional cases by PAEP immunostain identified a case (Fig. 6) in which the microsatellite overexpressed PAEP, but also a small subclone within the primary melanoma, suggesting that this may be the clone of origin for the microsatellite. In case 2, the microsatellite overexpressed four markers of angiogenesis and four markers of vasculogenic mimicry (Fig. 3). We confirmed the histologic presence of vasculogenic mimicry in the microsatellite but not the primary melanoma. Vasculogenic mimicry has been reported in melanoma previously [38–41]; however, our findings in case 2 suggest that it can be a key step in the evolution of microsatellite disease. Further study is necessary in this regard.

## Limitations and future studies

The conclusions of this study are limited by sample size. It would be desirable to validate expression of additional genes at the protein level by immunohistochemistry and also to validate the differentially expressed genes identified in functional studies. A limitation of this study is that the differentially expressed genes identified may not be functionally relevant to the evolution of microsatellite disease from primary melanoma. We have minimized this risk by focusing on genes that are highly differentially expressed between matched microsatellite and primary melanoma and also recurrent. Furthermore, we have focused on those genes that already have a significant literature demonstrating function in melanoma. An additional limitation is that microsatellites represent the end result of several stages in tumor evolution since they are thought to require vascular invasion, limited survival in a vascular space, exit from the vascular space, and proliferation in a distant tissue site. Vascular invasion is also present in FFPE sections adjacent to primary melanomas and likely represents a smaller increment in melanoma progression since the cells have not yet undergone exit from the circulation or proliferation at a distant site. It is likely more amenable to study by higher resolution spatial transcriptomic methods such as Visium HD, and we propose to examine this in future studies. A further limitation for studies such as this is that for many recurrently overexpressed genes there is currently limited understanding of the mechanism of their potential contribution to melanoma metastasis.

## Conclusion

This study is the first molecular characterization of melanoma microsatellites, and has identified overexpressed and under-expressed genes that mediate tissue remodeling, lymphovascular invasion, survival in the circulation, exit from the circulation, proliferation at a distant site, and immune evasion. The approach can be applied to any tumor that features microsatellites, and using higher resolution spatial transcriptomics platforms can be applied to smaller increments in tumor evolution such as vascular invasion.

### Methods

Cases of primary cutaneous melanoma with an adjacent microsatellite with available formalin-fixed paraffin embedded (FFPE) tissue were selected from the archives at Indiana University. The histologic diagnosis was confirmed by two board-certified dermatopathologists. Primary melanomas with their adjacent microsatellites were studied on the Visium platform (10 × Genomics). The Visium platform provides whole transcriptome sequencing of 55-µm diameter areas of tissue, suitable for studying primary melanoma and making comparisons to microsatellites in the same tissue section. FFPE sections were cut and placed on 6.5 mm Visium capture areas per the manufacturer’s protocol. Sequencing was performed on the Illumina platform, processed with Space Ranger and transferred to Loupe Browser for analysis. Twelve primary cutaneous melanomas with an available microsatellite were studied by immunohistochemistry. Immunohistochemistry for PAEP was performed using polyclonal Rabbit Ab HPA029473 (Millipore Sigma) at a dilution of 1:100 with 15 min of high pH heat-induced epitope retrieval, using the Vectastain Elite ABC-HRP kit (Vector labs) for color development. Immunohistochemistry for E-cadherin (clone 36, Ventana, prediluted), leukocyte common antigen (Clone RP2/18, Ventana, prediluted), and CD31 (clone JC70, Ventana, prediluted) was performed on the Ventana Benchmark Ultra platform. CD31 was counterstained manually with PAS. This study was conducted under an institutional IRB and did not require ethical approval.

## Supplementary Information

Below is the link to the electronic supplementary material.

Supplementary file 1 (PDF 2.53 MB)

## Data Availability

Data available on request from corresponding author.

## Abbreviations

DEG: Differentially expressed genes
FFPE: Formalin-fixed paraffin embedded
