# Supplementary material for: Melanoma microsatellites exhibit a metastatic signature by spatial transcriptomics and overexpress mediators of immune evasion
Source: Virchows Arch. 2025 Jul 28;487(6):1391–400. doi: 10.1007/s00428-025-04182-7 (PMC12748302; doi:10.1007/s00428-025-04182-7)
Supplement: Supplementary file 1 — (PDF 2.53 MB) [file 428_2025_4182_MOESM1_ESM.pdf]

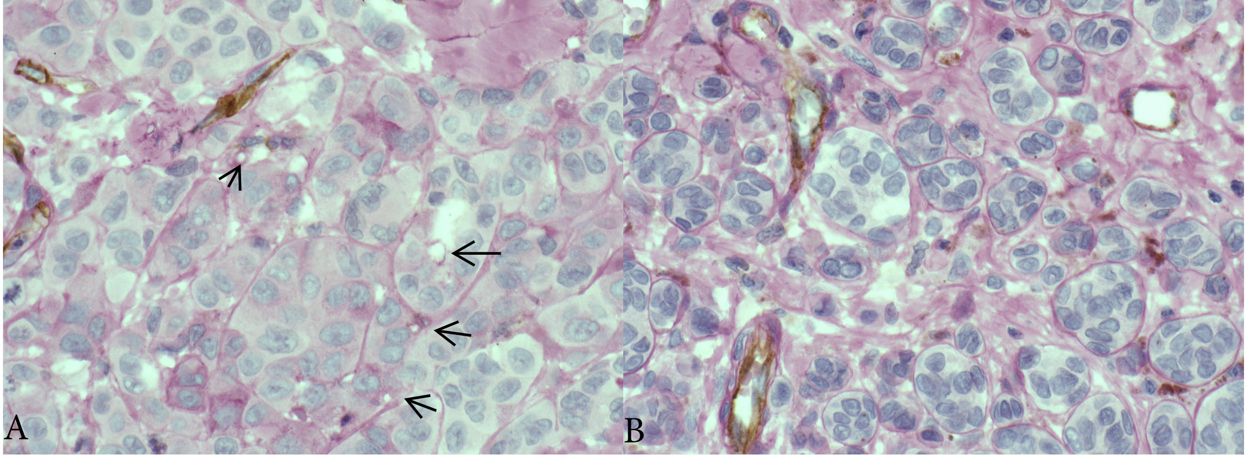

Figure S1. Combined CD31/ PAS stain shows vasculogenic mimicry is present in the microsatellite in case 2 but not its primary. A: Case 2, microsatellite. Normal capillaries are stained brown by CD31 component. Tubules of vasculogenic mimicry stain pink (arrows), 600x. B, Case 2 Primary melanoma. Normal capillaries are stained brown by CD31 component. No vasculogenic mimicry identified. 600x magnification.

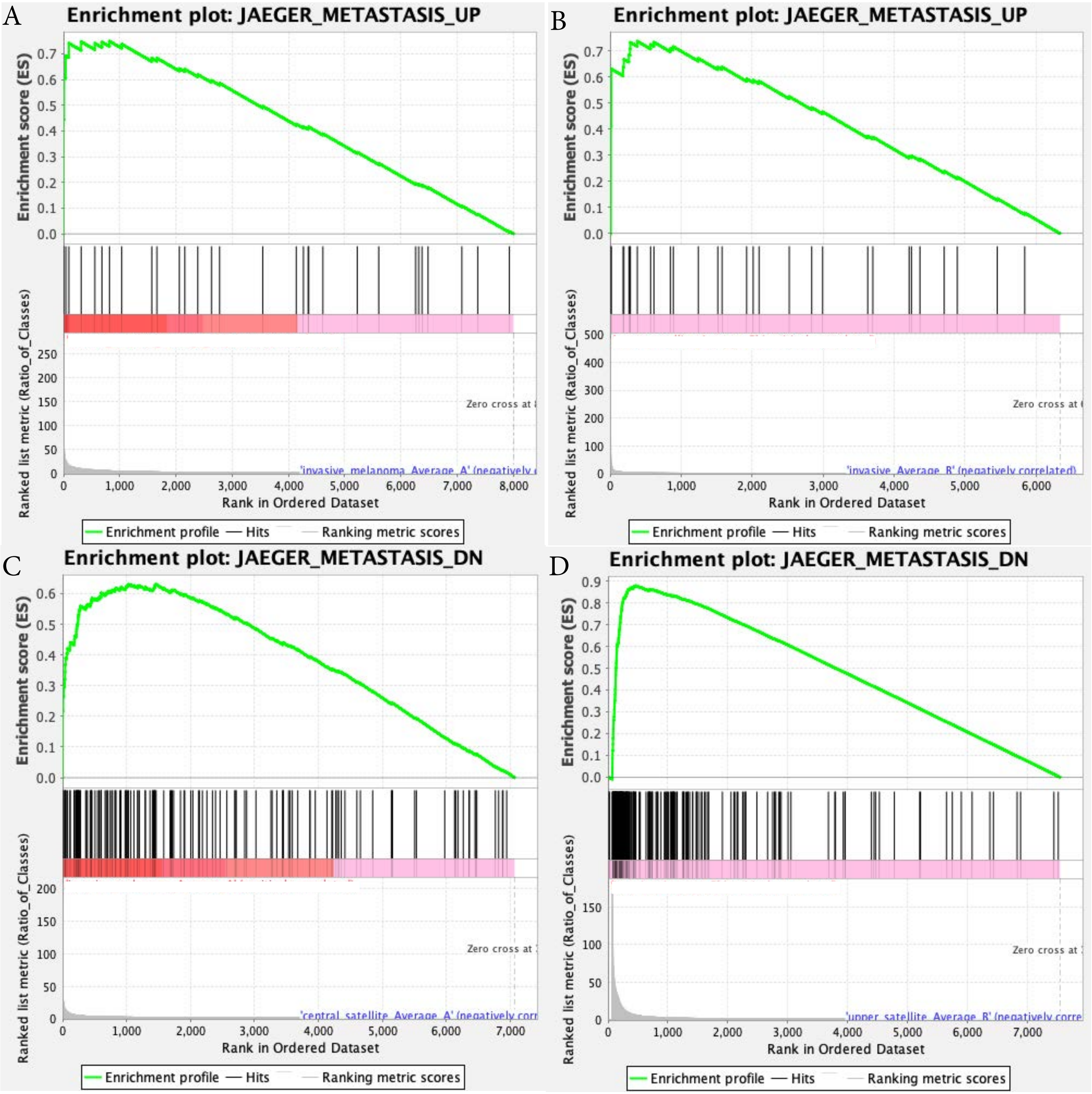

Figure S2. Gene set enrichment analysis identified significant enrichment for both up-regulated and down-regulated genes in melanoma microsatellites compared to a set of 22 melanoma distant metastases and 19 primary melanomas. A, B: Upregulated genes in cases 1, 2. $p=0.003$, $p=0.003$ C, D: Downregulated genes in cases 1, 2. $p=0.0$, $p=0.0$

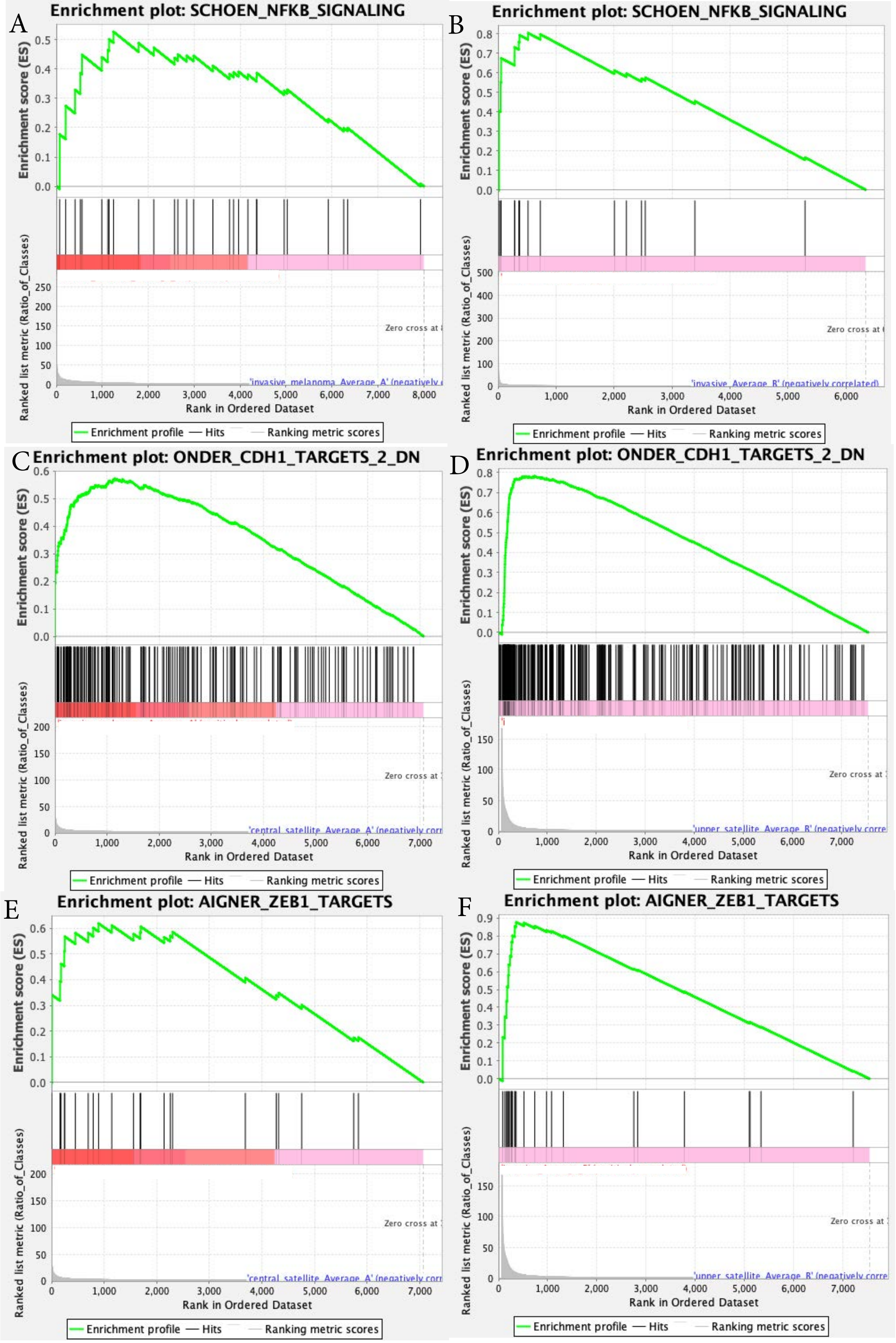

Figure S3. Gene set enrichment analysis identified recurrent changes in master regulator pathways NFKB, CDH1 and ZEB1 in melanoma microsatellites. A, B: Cases 1,2. Pathway enrichment for NFKB signaling in genes over-expressed in microsatellites. Case 2 was significant p=0.011 and case 1 was not significant p=0.11. C, D: Cases1, 2. Pathway enrichment for CDH1 targets in genes under-expressed in microsatellites Case1: p=0.0 , Case2: p=0.0.

E, F: Cases 1, 2. Pathway enrichment for ZEB1 targets in genes under-expressed in microsatellites Case1:p=0.03, Case2: p=0.0.
